# MK-8776 and Olaparib Combination Acts Synergistically in Hepatocellular Carcinoma Cells, Demonstrating Lack of Adverse Effects on Liver Tissues in Ovarian Cancer PDX Model

**DOI:** 10.3390/ijms26020834

**Published:** 2025-01-20

**Authors:** Wiktoria Bębenek, Arkadiusz Gajek, Agnieszka Marczak, Jan Malý, Jiří Smejkal, Małgorzata Statkiewicz, Natalia Rusetska, Magdalena Bryś, Aneta Rogalska

**Affiliations:** 1 Department of Medical Biophysics, Institute of Biophysics, Faculty of Biology and Environmental Protection, University of Lodz, 90-236 Lodz, Poland; wiktoria.bebenek@edu.uni.lodz.pl (W.B.); arkadiusz.gajek@biol.uni.lodz.pl (A.G.); agnieszka.marczak@biol.uni.lodz.pl (A.M.); 2 Doctoral School of Exact and Natural Sciences, University of Lodz, Jana Matejki 21/23, 90-237 Lodz, Poland; 3 Faculty of Science, University of Jan Evangelista Purkyně in Ústí nad Labem, 400 96 Ustí nad Labem, Czech Republic; jan.maly@ujep.cz (J.M.); jiri.smejkal@ujep.cz (J.S.); 4 Department of Genetics, Maria Sklodowska-Curie National Research Institute of Oncology, 5 Roentgena Street, 02-781 Warsaw, Poland; malgorzata.statkiewicz@pib-nio.pl; 5 Department of Experimental Immunology, Maria Sklodowska-Curie National Research Institute of Oncology, 5 Roentgena Street, 02-781 Warsaw, Poland; natalia.rusetska@pib-nio.pl; 6 Department of Cytobiochemistry, Institute of Biochemistry, Faculty of Biology and Environmental Protection, University of Lodz, 90-236 Lodz, Poland; magdalena.brys@biol.uni.lodz.pl

**Keywords:** liver cancer, ovarian cancer, metastasis, targeted therapy, olaparib, CHK1 inhibitor, replication stress

## Abstract

Hepatocellular carcinoma (HCC) cells critically depend on PARP1 and CHK1 activation for survival. Combining the PARP inhibitor (PARPi) olaparib with a CHK1 inhibitor (MK-8776, CHK1i) produced a synergistic effect, reducing cell viability and inducing marked oxidative stress and DNA damage, particularly in the HepG2 cells. This dual treatment significantly increased apoptosis markers, including γH2AX and caspase-3/7 activity. Both HCC cell lines exhibited heightened sensitivity to the combined treatment. The effect of drugs on the expression of proliferation markers in an olaparib-resistant patient-derived xenograft (PDX) model of ovarian cancer was also investigated. Ovarian tumors displayed reduced tissue growth, as reflected by a drop in proliferation marker Ki-67 levels in response to PARPi combined with CHK1i. No changes were observed in corresponding liver tissues using Ki-67 and pCHK staining, which indicates the absence of metastases and a hepatotoxic effect. Thus, our results indicate that the dual inhibition of PARP and CHK1 may prove to be a promising therapeutic approach in the treatment of primary HCC as well as OC tumors without the risk of liver metastases, especially in patients with olaparib-resistant tumor profiles.

## 1. Introduction

Olaparib is the first-in-class approved poly(ADP-ribose) polymerase (PARP) inhibitor (PARPi) indicated as monotherapy for the maintenance treatment of recurrent platinum-sensitive ovarian cancer (OC), regardless of *BRCA1/2* status [1]. At least 59% of ovarian cancers metastasize to other organs, with the liver, lungs, bone, and brain being the most common sites of metastasis. The 5-year survival rates for patients with metastases were 34% (liver), 20.4% (lung), 15% (bone), and 10% (brain) [2]. Among these, patients with liver metastases had better prognoses compared to other distant metastasis sites [3].

The liver is the sixth most common site of primary cancer and the fourth leading cause of cancer-related deaths worldwide. Hepatocellular carcinoma (HCC) accounts for 90% of all liver cancers [4]. Each year, there are approximately 840,000 new cases of HCC and at least 780,000 deaths globally [5]. Due to the lack of effective treatments, the prognosis for HCC remains poor, with an average 5-year survival rate of less than 10%. For patients with intermediate-stage HCC, transarterial chemoembolization (TACE) is standard treatment. This procedure involves the direct administration of doxorubicin or cisplatin into the arteries supplying blood to the tumor, followed by embolic agents such as a gelatin sponge to limit blood access to the tumor [6]. Patients with advanced HCC receive targeted therapies, including sorafenib, lenvatinib, regorafenib, ramucirumab, and cabozantinib. These therapies provide survival benefits but are often limited by the development of drug resistance [7]. Recent data indicate that patients with liver cancer carrying a *BRCA2* germline mutation may benefit from combination treatment involving olaparib. A case report showed stable disease and improvement in hepatalgia for 3 months after olaparib and nivolumab treatment [8]. *BRCA* genes encode proteins critical for the homologous recombination repair of DNA double-strand breaks. The accumulation of DNA damage, in the absence of functional repair mechanisms, leads to cell cycle arrest and, potentially, apoptosis. While significant progress has been made in targeting BRCA-mutated cancers, novel therapeutic strategies are urgently needed to counter this lethal disease. Olaparib is effective for ovarian cancer, irrespective of *BRCA* mutation or homologous recombination deficiency (HRD) status [9]. However, the effects of combining olaparib with replication stress inhibitors in liver cancer remain poorly understood. Recent studies have identified CHK1 signaling as a promising therapeutic target for liver cancer. CHK1 is a serine/threonine kinase. Therefore, CHK1 inhibition renders cells more sensitive to DNA damage and programmed cell death [10]. Two of these inhibitors, MK-8776 and prexasertib, have progressed to phase I/II clinical trials and demonstrated safety profiles and pharmacokinetic characteristics. The inhibitors affect CHK1 autophosphorylation at Ser296, therefore promoting CHK1 ubiquitylation and proteasomal degradation [11,12]. MK-8776 is approximately 500 times more selective for CHK1 than for CHK2 [13]. Moreover, MK-8776 has demonstrated the ability to sensitize p53-defective human tumor cells to DNA-damaging agents, enhancing the efficacy of gemcitabine (NCT00779584) or cytarabine (NCT01870596) treatment. This sensitization was achieved by abrogating the S-phase checkpoint, leading to increased DNA damage and cell death in cancer cells [10,14]. The inhibition of CHK1 has been shown to suppress proliferation in liver cancer cells having a high basal level of replication stress [15]. Thus, the aim of our studies was to demonstrate that the combination of olaparib and MK-8776 could be an effective treatment strategy for patients with a primary liver tumor. Initial in vitro studies confirmed the effectiveness of the combined therapy. We also investigated whether the drugs induced liver damage. Furthermore, we confirmed that the combination could be applicable as a second-line treatment for patients resistant to olaparib and who had developed liver metastases after first-line therapy. Our in vivo revealed no changes in liver tissues after treating olaparib-resistant PDX tumors.

## 2. Results

### 2.1. The Combination of the CHK1 Inhibitor and Olaparib Demonstrated a Synergistic Increase in Cytotoxicity Against HCC Cells, with No Enhancement Observed in PBMC Cells

**Figure 1:**
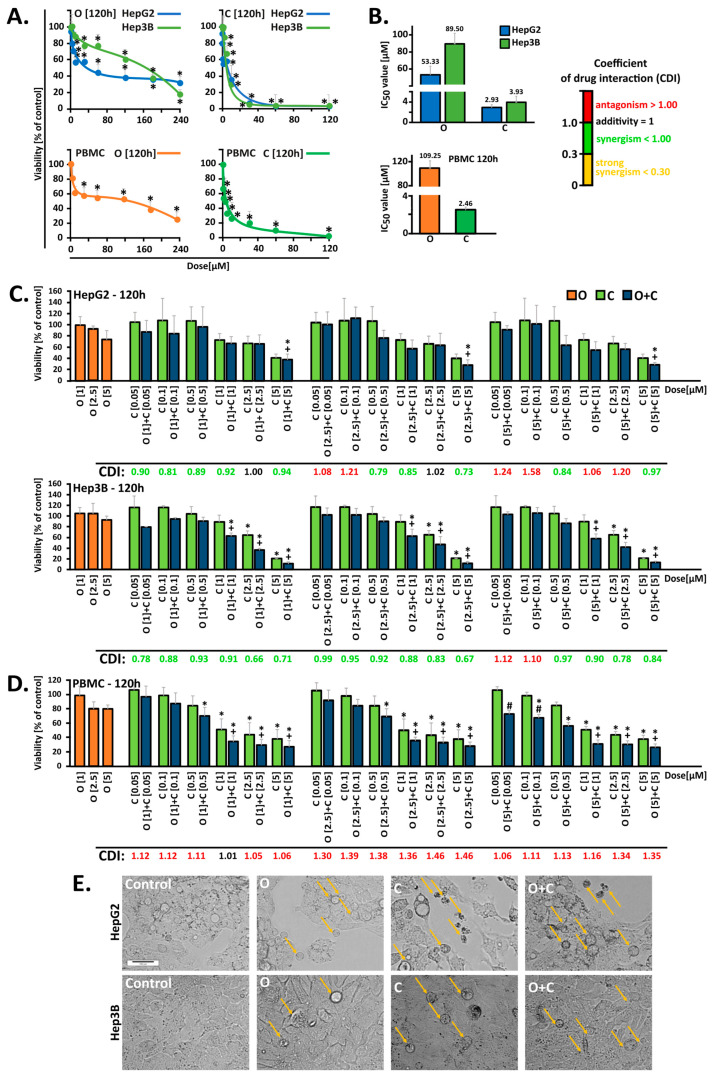
PARPi combined with CHK1i induces lower cytotoxicity in HepG2 cells compared to Hep3B cells. (**A**) Cell viability in response to five-day treatment with PARPi (O, 1–240 µM) and CHK1i (C, 0.05–120 µM) was determined using MTT and resazurin reduction assays in hepatocellular carcinoma and PMBC cells, respectively. (**B**) The IC_50_ values. Data are expressed as mean ± SD (n ≥ 3). (**C**,**D**) Cytotoxic effects observed in hepatocellular carcinoma and PBMC cells (120 h) after treatment with O (1, 2.5, or 5 µM) combined with C (0.05, 0.1, 0.5, 1, 2.5, and 5 µM). (**E**) Morphological changes in HCC cells in response to O (2.5 µM) and C (5 µM), or their combinations after five days of treatment. Images were captured at 10× magnification. Orange arrows show morphological changes. Data are expressed as mean ± SD (n = 3–6). Coefficient of drug interaction (CDI) values, indicating whether interaction effects are significantly synergistic (CDI < 1), additive (CDI = 1.0), or antagonistic (CDI > 1.0). Statistical significance was assessed using ANOVA followed by Tukey’s test. * Statistically significant changes between cells treated with the compound and control cells (*p* < 0.05). + Statistically significant changes between cells treated with PARPi and combination treatments (O + C) (*p* < 0.05). # Statistically significant changes between cells treated with CHK1i and combination treatments (O + C) (*p* < 0.05).

To evaluate the potential antitumor activities of PARPi, CHK1i, and their combinations, the cytotoxic effects of the inhibitors in liver cancer cell lines were investigated using an MTT assay. Dose–response curves were generated for all cell lines to estimate the IC_50_ values using a five-day treatment of cells with increasing concentrations of PARPi (1–240 μM) and CHK1i (0.05–120 μM) (Figure 1A). The efficacy of both PARPi and CHK1i was lower in the case of the Hep3B line compared to the HepG2 line. A reduction in the survival of HepG2 cells to 50% was noted at a 53.33 µM concentration of PARPi, while in the case of the Hep3B cells, a comparable reduction was observed at 89.5 µM (Figure 1B).

We further examined the hypothesis that PARPi cytotoxicity could be potentiated by the concurrent addition of CHK1i, promoting blockage of the DNA repair pathway. Specific concentrations of PARPi (1, 2.5, and 5 μM) and CHK1i (0.05, 0.1, 0.5, 1, 2.5, and 5 µM) were selected for additional analyses to determine the types of drugs interactions (Figure 1C). The lowest doses of PARPi and CHK1i that demonstrated synergistic effects in HepG2 and Hep3B cells were 2.5 μM and 55 µM, respectively. Combination treatment resulted in a 28% and 10.7% survival reduction in HepG2 (CDI = 0.73) and Hep3B (CDI = 0.67) cells, respectively. These changes were significantly higher than those with PARPi or CHK1i monotherapy. Moreover, the morphology of HepG2 and Hep3B cells was altered in response to both inhibitory agents tested after a five-day incubation period, as indicated by the spherical cell shape, loose adherence, or detachment from the surface, frequently accompanied by cell fragmentation (Figure 1E).

The dose–response curves and synergistic cytotoxic effects of the drugs were also determined with the resazurin reduction assay in response to 48 h (Figure S1) and 120 h of treatment in PBMC cells (Figure 1D). The estimated IC_50_ values after five days of treatment exhibited a notable decline in comparison to the elevated values observed after 48 h. PARPi was significantly less toxic to PBMCs compared to HCC cells. CHK1i, on the other hand, showed comparable cytotoxicity. Notably, selected concentrations of the compounds, administered in combination, did not exhibit greater cytotoxicity than that observed against HCC cells.

### 2.2. Oxidative Stress Generated by Olaparib and CHK1i Combination Differs Between HepG2 and Hep3B Cells

**Figure 2:**
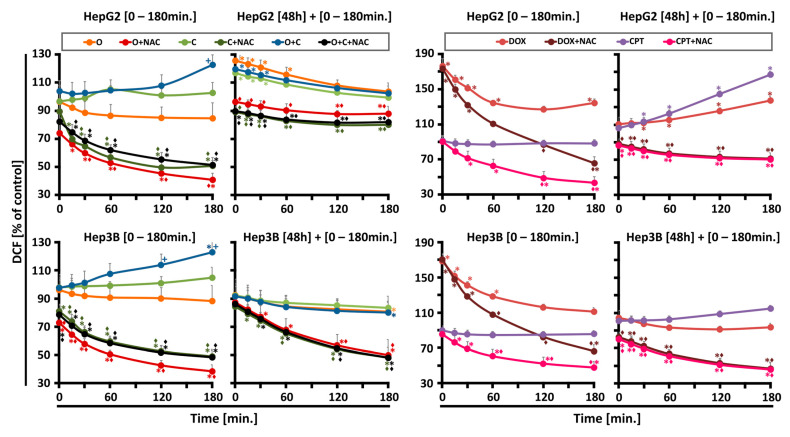
The kinetics of ROS generation in Hep3B and HepG2 cells after treatment with O (2.5 µM), C (5 µM), and the combination of O + C (2.5 µM; 5 µM) were measured immediately after adding the drugs up to 180 min, and after 48 h up to 180 min in the presence or absence of an antioxidant (NAC). The control cells (not treated) were assumed as 100%. Positive controls cells were treated with doxorubicin (DOX, 10 µM) or camptothecin (CPT, 5µM). Data are expressed as mean ± SD (n = 3–6). Statistical significance was assessed using ANOVA followed by Tukey’s test. * Statistically significant changes between cells treated with the compound compared with control cells (*p* < 0.05). + Statistically significant changes between cells treated with PARPi and combination treatments (PARPi/CHK1i) (*p* < 0.05). ◊ Statistically significant changes between cells treated with compound and samples preincubated with N-acetylcysteine (*p* < 0.05).

The objective was to examine the amount of ROS produced by PARPi, CHK1i, or their combinations. The extent of ROS production was found to be dependent on both the cell line and the duration of drug incubation. The initial measurement indicated that the cellular DCF fluorescence intensity was observed to be lower in cells incubated with each drug individually, in comparison to the drug combination (Figure 2). After 180 min of incubation, ROS production increased the most, with the fluorescence probe intensity rising by about 20% in both the Hep3B and HepG2 cells after PARPi + CHK1i treatment. Extending the incubation period to 48 h revealed a secondary rise in ROS levels exclusively in the HepG2 line, which was followed by a decline after 60 min of measuring the fluorescence probe intensity. This may suggest that intracellular antioxidant systems neutralize ROS.

The analysis of the level of superoxide anion radicals in the HepG2 and Hep3B cell lines was performed using the MitoSOX Red probe (Thermo Fisher Scientific, Waltham, MA, USA). In both tested cell lines, no statistically significant changes in the level of superoxide anion radicals were observed for all the tested compounds administered both in monotherapy and in combination, regardless of the incubation time (Figure S2).

### 2.3. CHK1i Monotherapy and Combination with PARPi Increases Apoptosis and DNA Damage

**Figure 3:**
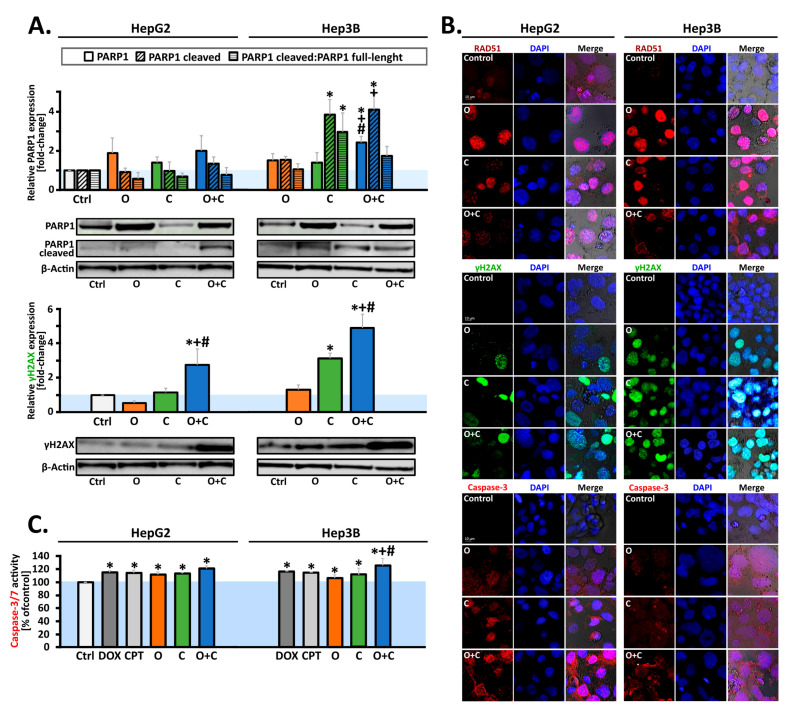
(**A**) Quantitative Western blot analysis and representative images of γH2AX, and full-length and cleaved PARP1 in HCC cells. Protein levels following treatment were quantified, with normaization to β-actin as a loading control and calculated as fold-change relative to untreated controls. (**B**) Representative images of immunofluorescence staining. HCC cells were incubated with O and C individually or O + C combination for 48 h and labeled with fluorochrome-conjugated antibodies against γH2AX (green fluorescence), RAD51, and caspase-3 (red fluorescence). Images were acquired using a confocal laser scanning microscope (magnification 63×). (**C**) Caspase-3/7 activity changes in HCC cells after single (O, C) or combined (O + C) drug administration. (Data are expressed as mean ± SD (n = 3–6). Statistical significance was assessed using ANOVA followed by Tukey’s test. * Statistically significant changes between cells treated with the compound compared with control cells (*p* < 0.05). + Statistically significant changes between cells treated with PARPi and combination treatments (PARPi/CHK1i) (*p* < 0.05). # Statistically significant changes between cells treated with CHK1i and combination treatments (PARPi/CHK1i) (*p* < 0.05).

We hypothesized that the inhibition of PARP1 and blockage of the CHK1 pathway could trigger elevated phosphorylation of H2AX, resulting in significant γH2AX formation in HCC cells. The proficiency and recruitment of HR repair factors with PARPi monotherapy were insufficient to significantly increase histone γH2AX expression in both studied HCC lines. However, the combined administration of inhibitors proved effective, significantly increasing the relative level of histone γH2AX expression in both lines, with this effect being almost twice as high in the Hep3B line (Figure 3A). The in situ confocal laser scanning immunofluorescence (Figure 3B) results were consistent with the Western blot data for cleaved γH2AX expression. Moreover, PARPi monotherapy was insufficient to significantly increase the PARP1 expression in both HCC cells. In the Hep3B line, both CHK1i monotherapy and the combined administration of CHK1i and PARPi significantly increased the level of cleaved PARP1 compared to the control. However, no statistically significant changes were observed in the HepG2 line. The combination of CHK1i and PARPi also reduced Rad51 foci formation, which may translate into increased PARP1 cleavage and heightened caspase 3/7 activity. Overall, we demonstrated that combining PARPi with CHK1i induced greater DNA damage in HCC cell lines compared to either PARPi or CHK1i monotherapy. Because DNA damage induces apoptosis, we investigated the effect of the compounds on the caspase 3/7 ratio, a marker of apoptosis. The results are presented in Figure 3B,C. In the Hep3B line, the combination significantly increased the level of caspase 3 compared to monotherapy. In the HepG2 line, the changes were lower, which translated into a statistically insignificant increase in cleaved PARP1.

### 2.4. HCC Cells Display Differences in Native ALT Activity and Susceptibility to PARP and CHK1 Inhibitors

**Figure 4:**
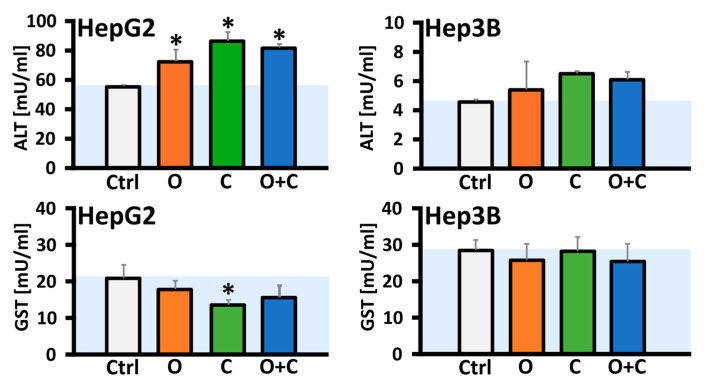
O combined with C induced alanine aminotransferase activity and glutathione S-transferase after 48 h of incubation in HCC cells. Data are expressed as mean ± SD (n = 3–6). Statistical significance was assessed using ANOVA followed by Tukey’s test. * Statistically significant changes between cells treated with the compound compared with control cells (*p* < 0.05).

Our study revealed that in the HepG2 cells, the ALT level in the control was almost 12 times higher than in the Hep3B cell line. Both PARPi and CHK1i monotherapy and the combination of these two inhibitors in HepG2 cells caused a statistically significant elevation of alanine aminotransferase activity above the control cells. In contrast, treatment of Hep3B cells with the same compounds did not result in statistically significant alterations in ALT activity. Moreover, exposure of HepG2 cells to the tested inhibitors resulted in a notable reduction in glutathione S-transferase activity relative to control cells, with the most pronounced effect observed following CHK1i monotherapy. In the Hep3B cells, PARPi monotherapy and the combined treatment resulted in a slight but statistically insignificant reduction in enzyme activity. Conversely, CHK1i monotherapy was observed to maintain glutathione S-transferase activity at levels comparable to the control (Figure 4).

### 2.5. Combined Antitumor Activity of PARPi and CHK1i: Histopathological Analysis of Tumor and Liver Tissues

**Figure 5:**
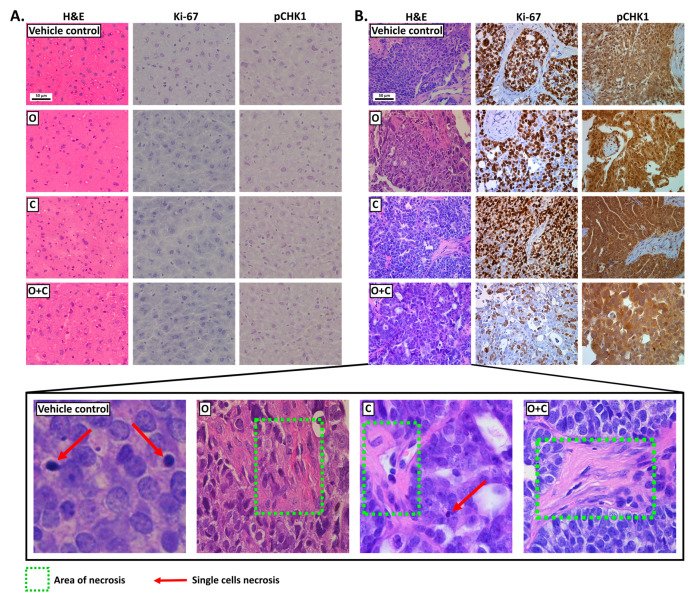
Characterization of responses of liver tissues (**A**) and ovarian tumors (**B**), collected from mice with olaparib-resistant PDX tumors, to the tested inhibitors based on a histopathological assessment (H&E-staining) and evaluation of Ki-67 and pCHK1 expression levels (IHC staining). Images were captured at 20× magnification, MICA WideFocal Live Cell, Leica Microsystems, Wetzlar, Germany).

To evaluate the effectiveness and side effects of the drugs in patient-derived xenograft (PDX) models, we examined whether ovarian tumor cells metastasized to the liver after therapy. Olaparib-resistant PDX tumors were treated with PARPi alone or in combination with a CHK1 inhibitor for histopathological analysis. The levels of Ki-67 (a proliferation marker) and CHK1 kinase phosphorylation at Ser345 were assessed (Figure 5). Detailed results of the tumor morphology analysis, including the location, nature, and severity of pathological changes in PDX tissues stained with H&E, were previously described [16].

Representative H&E-stained images of the liver tissues and morphological changes are presented in Figure 5. An H&E-based evaluation of the liver tissue from mice with olaparib-resistant ovarian cancer revealed no evidence of metastasis to the liver. In contrast, most drugs-treated ovarian tumors exhibited greater signs of necrosis, potentially associated with an increased growth rate. The evaluation of protein biomarkers, using IHC staining (Leica biosystems, Nussloch, Germany), revealed a significant decrease in Ki-67 levels in the cohort treated with the combination of PARPi and CHK1i. Additionally, Ki-67 and pCHK staining showed no changes in liver tissue, indicating the absence of metastases and confirming that the treatment did not induce hepatotoxic effects.

## 3. Discussion

Hepatocellular carcinoma (HCC) is one of the most common solid tumors, and despite significant advancements in medical technology, effective therapeutic options with curative potential remain limited for patients with liver cancer. One promising target for treatment is the increased genomic instability observed in HCC. Highly proliferating liver cancer cells must manage substantial DNA damage induced by replication stress and heightened oxidative stress. For in vitro studies on liver cancer, the HepG2 and Hep3B cell lines are frequently utilized. Although both are common research models, they exhibit key differences. HepG2 expresses the majority of drug-metabolizing enzymes, making it a relevant model for pharmacological studies. Furthermore, HepG2 lacks the hepatitis B virus (HBV) genome, whereas Hep3B harbors integrated HBV sequences, a distinction of particular relevance since HBV infection is strongly associated with liver cancer [17]. These differences can lead to divergent and sometimes opposing responses to the same pharmacological treatments under identical experimental conditions. This variability underscores the importance of selecting the appropriate cell line for specific research objectives, particularly in studies on liver cancer treatment strategies. Moreover, HBV-infected patients with liver cancer might also carry a *BRCA2* germline mutation, which further complicates treatment responses [8].

The increase in homologous recombination (HR) efficiency resulting from the rapid proliferation of liver cancer cells causes high replication stress. This stress can be alleviated by HR, making the combined use of PARPi and a CHK1 inhibitor (CHK1i) a potential therapeutic strategy. This approach could benefit not only patients with primary liver cancer but also those with liver metastases from ovarian cancer.

In the initial stage of our investigations, we conducted in vitro studies on HCC and PBMC cells to evaluate the therapeutic potential of the drugs, both following individual and combined administration. Five-day monotherapy with PARPi at a concentration of 2.5 µM strongly reduced cell survival, decreasing it by up to 60% in HepG2 cells and 30% in Hep3B cells [18]. Other studies have shown that in HepG2 cells, a five-day incubation with 10 µM PARPi decreases survival to approximately 60% after 96 h of incubation. Similarly, a four-day incubation with PARPi at concentrations of 1, 3, and 10 µM caused a comparable reduction in survival across several liver cancer lines, including HepG2, Huh7, and SNU-398, with the effect being more pronounced in the HepG2 line. Interestingly, PARPi at these concentrations did not exhibit cytotoxic activity in liver cancer cells such as SNU-449 and PLC/PRF/5, highlighting the heterogeneity in drug response among liver cancer subtypes [19]. CHK1 has been identified as a potential therapeutic target for hepatocellular carcinoma treatment [10]. However, monotherapy with the CHK1 kinase inhibitor (MK-8776) and its combination with PARPi have not been previously tested in HCC models. In our study, the combination of MK-8776 and olaparib demonstrated an enhanced cytotoxic effect, as confirmed by the CDI values.

To evaluate off-target effects, PBMC cells isolated from buffy coats were used as a control model. Our studies demonstrated that the selected concentrations of compounds used in combination did not intensify the cytotoxic effects observed in HCC cells when applied to PBMC cells. The results obtained in in vitro studies, including those regarding PBMC survival, do not always reflect the actual effects of therapy in patients. Therefore, in clinical practice, the benefits of treating cancer may outweigh the potential side effects, which are monitored and managed to ensure the best possible treatment outcome, also taking into account the health and functioning of immune cells such as PBMC [6,20].

Unrepaired single-strand breaks (SSBs) resulting from oxidative damage are directed toward homologous recombination (HR), significantly contributing to PARPi toxicity. PARP1 also plays a critical role in regulating mitochondrial function and oxidative metabolism, indicating that repair of oxidative damage may alter PARPi toxicity profiles. Overall, our studies revealed that ROS generation was influenced by the drug combination, incubation time, and cell type, but superoxide levels remained unchanged. Initially, ROS production was lower for individual drugs compared to their combination, which after 180 min, resulted in the highest increase in ROS levels. After prolonged incubation, a secondary ROS rise occurred exclusively in HepG2 cells, followed by a decline after 60 min, suggesting antioxidant system activity.

Recent findings suggest that CHK1 acts as a nuclear hydrogen peroxide sensor, initiating a cellular program to suppress ROS. CHK1 phosphorylates mitochondrial SSBP1 to prevent its nuclear localization, thereby reducing H_2_O_2_ levels. Nuclear hydrogen peroxide (H_2_O_2_) oxidizes C408, a highly conserved cysteine within the C-terminal kinase associate 1 (KA1) domain of CHK1’s autoinhibitory region, thereby activating CHK1. Co-treatment with CHK1 inhibitors may disrupt this protective mechanism, enhancing tumor sensitivity [21].

DNA damage response (DDR) machinery plays a significant role in hepatocarcinogenesis and might be used as biomarkers for predicting the risk of HCC development [15]. PARP inhibitors promote the accumulation of double-strand DNA breaks, indicated by the phosphorylation of histone H2AX at serine 139 (γH2AX). In this study, we evaluated whether PARP1 inhibition, combined with blockade of the CHK1 pathway, enhances H2AX phosphorylation in HCC cells. Western blot analyses showed that the combined administration of CHK1i and PARPi significantly increased γH2AX expression in both tested cell lines compared to the respective monotherapies, with the effect being more pronounced in the Hep3B cells. It was also proven that PARPi monotherapy did not induce a significant increase in γH2AX expression in the SNU-398 and SNU-449 HCC cells [15]. These findings underscore the importance of tailoring therapy based on the liver cancer subtypes and highlight the need for biomarkers to identify patients who are likely to benefit from combination therapy [22]. Recent studies have revealed a correlation between a deficiency in Rad51 and sensitivity to the PARPi [23]. PARP1 inhibition is associated with the induction of Rad51 nuclear accumulation and focuses on sites of DNA damage for initiation of repair. The over-expression of Rad51 can provide resistance to DNA-damaging agents, which may partly explain the limited monotherapy activity of PARPi against HCC cells. Therefore, CHK1i is a reasonable target for a combination strategy with PARPi to maximize DDR inhibition and drive tumor cell death in HCC cells [24]. In both tested cell lines, the combination of PARPi and CHK1 inhibition almost completely prevented the formation of nuclear RAD51 foci. Similar reports revealed that dehydroxymethylepoxyquinomicin (DHMEQ) and DHMEQ–olaparib treatment decreased the number of Rad51 nuclear foci in Hep3B cells. These findings align with other studies, demonstrating a decrease in Rad51 nuclear foci through the synergistic effects of cisplatin and olaparib combinations [23].

To explore the mechanism of cell death driving the cytotoxic effects of CHK1i and PARPi, we analyzed the levels of apoptotic markers. The activity of caspase-3 and the cleaved-PARP1 levels were determined. The combined use of both inhibitors seems to be justified because caspase-2/3-dependent apoptotic responses are blocked by CHK1 independently of p53 [25]. Our results confirmed that CHK1i combined with PARPi treatment enhanced caspase-3 activation in Hep3B cells more significantly than in HepG2 cells. Additional evidence demonstrated that the combined use of olaparib and the DNA-PKcs inhibitor (NU7441) elevated caspase-3 levels in Hep3B cells [26], while treatment with two PARP inhibitors, AG014699 and BSI-201, increased the caspase-3 and -8 levels in HepG2 cells [27].

The activation of executive caspases, which is a consequence of programmed cell death, causes PARP1 proteolysis, during which the protein binding domain is separated from the catalytic domain [28]. Therefore, we also assessed the relative expression of cleaved PARP1 and the full-length protein. Our investigation demonstrated that the combination of both inhibitors elevated the expression of the full-length protein exclusively in the Hep3B cell line. Furthermore, in Hep3B cells, both CHK1i monotherapy and the combined treatment significantly increased the levels of cleaved PARP1 compared to the control. In contrast, no statistically significant changes were observed in the HepG2 cells following monotherapy or combined treatment, which may be attributed to a minimal increase in caspase-3/7 activity. Elevations in liver enzymes, particularly alanine aminotransferase (ALT), serve as biomarkers for potential liver dysfunction. For instance, elevated ALT levels in blood serum without significant increases in bilirubin may suggest hepatocellular damage, which could include cancer development. Drug-induced liver injury (DILI) has been reported in only two cases in a woman treated with the PARP inhibitor olaparib, though the exact mechanism of damage remains unclear [29,30]. Recent research highlights the AST/ALT ratio as a readily accessible and novel prognostic factor for HCC patients undergoing thermal ablation combined with transartelial chemoembolization (TACE). This ratio may also serve as a marker for chemotherapy-induced toxicity [31]. In our study, the relative activity of ALT was evaluated in HCC cell lysates, revealing a significant increase in enzyme activity exclusively in the HepG2 cells following either individual or combined drug administration, with CHK1i monotherapy showing nearly a 30% increase compared to the control. In clinical contexts, a phase I trial (NCT00516724) reported a case of elevated ALT levels persisting for 8 days following olaparib (100 mg twice daily) in combination with carboplatin and paclitaxel, which led to discontinuation of olaparib [29]. Elevation of alanine and/or aspartate aminotransferases (ALT/AST) was also frequent with rucaparib in the ARIEL3 study, but it was mainly transient and self-limiting [30]. Increased ALT/AST levels were also reported in early-phase studies of the WEE1 inhibitor (adavosertib), but not with elimusertib and prexasertib (CHK1i) [32,33]. Another biomarker of liver neoplasms is glutathione S-transferase (GST), an enzyme critical for detoxification processes and resistance to anticancer drugs. GST helps protect cells from cytotoxic and carcinogenic agents, and higher GST expression is often correlated with better HCC outcomes. Conversely, reduced GST activity may suggest diminished detoxification capacity, potentially enhancing the efficacy of anticancer drugs [34]. In our study, a significant decrease was observed only after CHK1i monotherapy in the HepG2 cells. However, it is important to highlight that conflicting data exist regarding the levels of GST in oncologic patients. In the study by Su et al., 2003 [35], an increased level of GST was found in patients with breast cancer compared to the control group, while other data showed reduced GST levels in breast cancer patients compared to healthy controls [36].

To further clarify the safety and impact of CHK1i and PARPi on the normal liver following treatment of PDX models of HGSOC, which have the potential to metastasize to the liver, we performed a histopathological analysis. This included evaluating the expression of Ki-67 (proliferation marker) and assessing CHK1 kinase activation through phosphorylation at Ser345. The histopathological analysis of liver tissues from randomly selected mice after treatment showed no signs of hepatocyte degeneration from the vehicle, PARPi, or CHK1i, either alone or in combination. Additionally, an evaluation of the protein biomarkers by IHC staining disclosed a lack of Ki-67 and pCHK1 expression in all liver tissue samples compared to the corresponding tumor samples. The difference in marker expression profiles demonstrates the absence of tumor foci in the livers of mice with induced ovarian cancer following treatment with PARPi and CHK1i. The use of compounds capable of inducing genetic instability in HCC cells appears highly promising. Notably, the combination of these compounds in ovarian cancer therapy did not trigger a harmful DILI reaction in the healthy livers of mice, nor did it lead to an excessive increase in cell proliferation. On the other hand, targeted therapy for liver cancer has been shown to be effective in vitro and will require further confirmation in preclinical studies.

## 4. Materials and Methods

### 4.1. Chemicals

PARP inhibitor (O, PARPi, Olaparib, AZD2281) and CHK1 inhibitor (C, CHK1i, MK-8776) were purchased from Selleck Chemicals (Houston, TX, USA). The inhibitor stock solutions were prepared in 100% dimethyl sulfoxide (DMSO) and stored at −80 °C. DMEM and RPMI 1640 culture media, heat-inactivated fetal bovine serum (FBS), and trypsin-EDTA were obtained from Gibco (Thermo Fisher Scientific, Waltham, MA, USA). The other essential reagents employed in the studies are described in Section 4 and listed in the Supplementary Tables S1 and S4.

### 4.2. Cell Culture

Human hepatocellular carcinoma HepG2 and Hep3B cell lines were purchased from ATCC (Rockville, MD, USA) and cultured as monolayers in high-glucose DMEM culture medium, which included GlutaMAX supplement and HEPES and was enriched with 10% FBS. The cells were cultured at 37 °C in a 5% (*v*/*v*) CO_2_ incubator. In parallel, the cytotoxicity test was performed on Peripheral Blood Mononuclear Cells (PBMCs). The blood buffy coat used to isolate these cells was obtained from the Blood Bank in Lodz (Lodz, Poland).

PBMCs were isolated from human blood buffy coat using Lymphosep (Biowest, Bradenton, FL, USA) density gradient centrifugation. Blood samples were first centrifuged at 1620× *g* for 10 min at 22 °C. The leukocyte-platelet buffy coat was collected, diluted with PBS containing EDTA, layered onto a Lymphosep gradient, and centrifuged again under the same conditions for 30 min. The resulting lymphocyte-platelet ring was lysed to remove erythrocytes (Table S3), followed by three washes in PBS without EDTA. The cells were then counted and resuspended in RPMI 1640 medium supplemented with 10% FBS, 1% antibiotic, and 1% phytohemagglutinin (Biowest, Bradenton, FL, USA) for further experiments.

### 4.3. MTT Cell Viability Assay

The cytotoxic effects of the drug and its influence on cell viability, which is closely tied to the metabolic activity of the cells, were assessed using the MTT assay. HepG2 or Hep3B cells were seeded into 96-well plates (5 × 10^3^ cells per well) in 100 μL of culture medium and incubated for 24 h at 37 °C with 5% CO_2_. The next day, each well received 50 μL of fresh culture medium along with 50 μL of 4 × concentrated drug dilutions prepared in the same medium. The cells were then incubated for five days at 37 °C with 5% CO_2_. After the treatment period, the medium was carefully removed, 50 μL of MTT solution (0.5 mg/mL in PBS) was added to each well, and the plates were incubated for 4 h at 37 °C with 5% CO_2_. Subsequently, 100 μL of DMSO was added to each well and incubated under the same conditions, until the formazan crystals were completely dissolved.

The absorbance was measured spectrophotometrically using a microplate reader (Synergy HTX, BioTek, Shoreline, WA, USA) at an experimental wavelength of 580 nm, with 720 nm as a reference wavelength. To assess cell survival, the absorbance at 720 nm was subtracted from the absorbance at 580 (A580-A720) for each individual well. Relative cell viability was calculated as the percentage of viability compared to untreated control cells. The MTT assay was used to plot dose–response curves for PARPi (1–240 μM) and CHK1i (0.05–120 μM) over a five-day period.

The overall cytotoxic impact of the simultaneous administration of two inhibitors over five days was evaluated by calculating the coefficient of drug interaction (CDI). This approach helped identify the lowest and most effective doses of PARPi (1, 2.5, and 5 μM) when combined with the CHK1 inhibitor (0.05, 0.1, 0.5, 1, 2.5, and 5 μM) for use in subsequent experiments. The CDI was computed using the formula CDI = AB/(A × B) [37]. In this formula, AB represents the ratio of the absorbance from the untreated control group, while A and B refer to the ratios of the single-agent groups to the control group. CDI values were used to categorize the interaction effects as synergistic (CDI < 1.0), additive (CDI = 1.0), or antagonistic (CDI > 1.0).

### 4.4. Cell Viability Assay Using Resazurin Reduction

PBMCs were seeded into black 96-well plates (1 × 10^5^ cells per well) in 100 μL of RPMI 1640 culture medium supplemented with 10% fetal bovine serum, 1% antibiotic, and 1% phytohemagglutinin. To each well, 50 μL of serially diluted compounds were added to achieve final concentrations ranging from 1 to 240 μM for PARPi and 0.05 to 120 μM for CHK1i, with a final well volume of 200 μL. The cells were incubated for 48 and 120 h at 37 °C with 5% CO_2_.

Following incubation, 30 μL of resazurin solution in PBS (final concentration 10 μg/mL) was added to each well. After 3 h incubation under the same conditions, the fluorescence of the resazurin reduction product, resorufin, was measured using a Fluoroskan Ascent FL microplate reader (Labsystem, Helsinki, Finland) with an excitation wavelength of λ = 530 nm and an emission wavelength of λ = 590 nm. For each well, the fluorescence measured at 530 nm was subtracted from the fluorescence measured at 590 nm. Relative cell viability was calculated as the percentage of viability compared to untreated control cells. This assay was used to plot dose–response curves for PARPi (1–240 μM) and CHK1i (0.05–120 μM) over two- and five-day periods. To analyze the drug interactions between PARPi combined with CHK1i, the coefficient of drug interaction (CDI) was calculated as described previously. PBMCs were isolated from human blood buffy coat using Lymphosep (Biowest, Bradenton, FL, USA) density gradient centrifugation. Blood samples were first centrifuged at 1620× *g* for 10 min at 22 °C. The leukocyte-platelet buffy coat was collected, diluted with PBS containing EDTA, layered onto a Lymphosep gradient, and centrifuged again under the same conditions for 30 min. The resulting lymphocyte-platelet ring was lysed to remove erythrocytes, followed by three washes in PBS without EDTA. The cells were then counted and resuspended in RPMI 1640 medium supplemented with 10% FBS, 1% antibiotic, and 1% phytohemagglutinin (Biowest, Bradenton, FL, USA) for further experiments.

### 4.5. Cellular Morphology Changes

The morphological changes induced by tested compounds in Hep3B and HepG2 cells were evaluated by the brightfield microscopy technique. Cells were seeded in 12-well plates (5 × 10^4^ cells) containing 1 mL of culture medium and incubated for 24 h (37 °C, 5% CO_2_). The following day, 0.5 mL of fresh culture medium was added to each well and the cells were treated for five days (37 °C, 5% CO_2_) with PARPi (2.5 µM), CHK1i (5 µM), and their combinations by adding 0.5 mL of 4 × concentrated working dilutions of drugs prepared in culture medium. Afterward, cellular morphology changes were imaged at 10× magnification using an inverted optical microscope (Olympus IX70, Tokyo, Japan).

### 4.6. Measurement of Intracellular and Mitochondrial ROS

HepG2 and Hep3B cells were seeded in black 96-well plates (7 × 10^3^ cells per well) in 100 μL of medium. After a 24 h incubation at 37 °C with 5% CO_2_, the medium was removed, and wells were washed with PBS. The plates were then divided into different treatment groups, each with and without an antioxidant (3 mM NAC, N-acetylcysteine). The treatment conditions included 2.5 μM, 5 μM, and a combination of PARPi + CHK1i. In our experiments, we also used 10 μM doxorubicin and 5 μM camptothecin (as positive controls).

Measurements were performed in two time variants, shortly and after 48 h of continuous incubation with drugs, for both probes (MitoSOX Red and H2DCF-DA) (Thermo Fisher Scientific, Waltham, MA, USA). In the short-term variant, fluorescent probes were added 30 min before the tested compounds. Then, the drugs, with or without NAC, were added simultaneously. Fluorescence was measured in the time range of up to 180 min until the ROS growth: immediately after adding the drugs and 15; 30; 60; 120; and 180 min. In the longer term, the plates were incubated for 48 h with the tested compounds at 37 °C in 5% CO_2_. Some of the cells were preincubated with an antioxidant NAC for 30 min; then, the drugs were added at the appropriate concentrations; and incubation was continued for the required period of time under the same conditions. Following the incubation period, the medium was removed, and each well was washed three times with PBS. Intracellular ROS generation was detected using H2DCF-DA. A fluorescent probe, 5 µM, was added to each well, followed by a 30 min incubation at 37 °C. The wells were then filled with RPMI 1640 medium without phenol red to a final volume of 200 μL. Fluorescence was measured using a Fluoroskan Ascent FL microplate reader (Labsystem, Helsinki, Finland) at an excitation wavelength of λ = 485 nm and an emission wavelength of λ = 538 nm.

Mitochondrial ROS activity was measured with MitoSOX Red assay, a redox-sensitive fluorescent probe that is selectively targeted to the mitochondria. A fluorescent probe, 5 µM, was added to each well, followed by 45 min incubation at 37 °C. The wells were then filled with RPMI 1640 medium without phenol red to a final volume of 200 μL. Fluorescence was measured using a Fluoroskan Ascent FL microplate reader (Labsystem, Helsinki, Finland) at an excitation wavelength of λ = 390 nm and an emission wavelength of λ = 590 nm.

### 4.7. Western Blotting

For analyses of protein expression levels, the HepG2 and Hep3B cells were seeded in 100 mm Petri dishes (1.5 × 10^6^ cells) and incubated for 24 h (37 °C, 5% CO_2_), followed by treatment with pre-selected doses of tested compounds or their combinations for two days (2.5 μM PARPi and 5 μM CHK1i). Whole-cell lysates were prepared on ice by washing the cells with ice-cold DPBS and scraping them into ice-cold RIPA buffer containing phenylmethylsulfonyl fluoride (1 mM PMSF), Halt Protease Inhibitor Cocktail, and Halt Phosphatase Inhibitor Cocktail. The lysates were collected in microcentrifuge tubes, subjected to sonication using a probe sonicator (15 s), centrifuged at 13,000× *g* for 10 min at 4 °C, and transferred to fresh tubes. Protein samples were prepared in mPAGE 4X LDS Sample Buffer with 10X β-mercaptoethanol and heated at 70 °C for 10 min, and 25 μg of protein from each sample was loaded per lane. Proteins were separated by SDS-PAGE at 200 V for approximately 30 min using 10% mPAGE Bis-Tris gels in an electrophoresis tank (Mini-PROTEAN Tetra Cell, Bio-Rad, Hercules, CA, USA) with MOPS SDS running buffer. The proteins were then transferred to 0.45 μm PVDF membranes using a semi-dry transfer system (Trans-Blot Turbo Blotting System, Bio-Rad) with mPAGE Transfer Buffer, following the manufacturer’s optimized protocol for mPAGE Bis-Tris gels. The membranes were blocked with 5% non-fat milk in TBST (Table S2) for 1 h and washed with TBST (3 × 5 min). Primary antibodies were applied overnight at 4 °C (Table S5), followed by incubation with appropriate HRP-conjugated secondary antibodies for 2 h at room temperature (Table S6).

Protein detection was carried out using the enhanced chemiluminescence phenomenon. Membranes were incubated for 5 min with a chemiluminescent substrate (SuperSignalTM West Pico PLUS, Thermo Fisher Scientific, Waltham, MA, USA), and the emitted light was measured using an Azure 300 reader (Azure Biosystems, Dublin, CA, USA) equipped with a CCD camera (Figure S3). A densitometric analysis of protein band intensities was performed using ImageJ software, version 1.45 (National Institutes of Health, Bethesda, MD, USA). The relative expression level of target proteins compared to the control (cells not treated with the test compounds) was determined after normalizing the results against the corresponding bands for β-actin.

### 4.8. Alanine Transaminase Activity Assay

The effect of the studied inhibitors and their combinations on the relative activity of the ALT enzyme (DILI marker) was determined using the Alanine Transaminase Activity Assay kit (Abcam, Cambridge, UK) according to the manufacturer’s protocols. HepG2 and Hep3B cells were seeded into 100 mm dishes (1 × 10^6^ cells) in 8 mL of medium, and incubated for 24 h (37 °C, 5% CO_2_). After 24 h incubation, the culture medium was removed and the cells were washed with PBS, followed by treatment with pre-selected doses of tested compounds or their combinations for two days (2.5 μM PARPi and 5 μM CHK1i). After incubation, the medium with compounds was removed, and the cells were washed with PBS. Trypsin-EDTA was added to each dish. After trypsinization, the cell suspension was collected into 15 mL Falcon tubes. The cells were adjusted to a density of 1 × 10^6^ cells/mL in Eppendorf tubes and centrifuged (13,000× *g* for 5 min at 25 °C). The supernatant was discarded, and the cells were resuspended in PBS, centrifuged again, and resuspended in 200 μL of ALT Assay Buffer. The lysates were sonicated for 15 s and centrifuged for 10 min at 13,000× *g* at 4 °C. The clear supernatant was used for further analysis. A standard curve was prepared on a black 96-well plate with a glass bottom, and fluorescence was measured at an excitation wavelength of λ = 530 nm and an emission wavelength of λ = 590 nm using a Fluoroscan Ascent FL microplate reader (Thermo Fisher Scientific, Waltham, MA, USA). Blank readings were subtracted from the fluorescence measurements. Sample values were recalculated based on linear regression equations, and the absorbance difference was computed, followed by further calculations as per the manufacturer’s instructions.

### 4.9. Glutathione S-Transferase Activity Assay

The effect of the studied inhibitors and their combinations on the relative activity of glutathione S-transferase was analyzed using the Glutathione S-Transferase Fluorescent Activity Kit (Invitrogen, Thermo Fisher Scientific, Waltham, MA, USA) according to the manufacturer’s protocols. HepG2 and Hep3B cells were seeded into 100 mm dishes (1.5 × 10^6^ cells) in 8 mL of medium and incubated for 24 h (37 °C, 5% CO_2_). After 24 h incubation the culture medium was removed and the cells were washed with PBS, followed by treatment with pre-selected doses of the tested compounds or their combinations for two days (2.5 μM PARPi and 5 μM CHK1i).

After incubation, the medium containing the compounds was removed, and the cells were washed with PBS. The cells were then treated with trypsin-EDTA for 3 to 5 min at 37 °C. Then, the cells were centrifuged (5 min at 500× *g* and 25 °C) and washed with PBS. The cell pellet was resuspended in PBS and subjected to centrifugation at 500× *g* and 25 °C. The supernatant was removed. Assay Buffer was added to the cell pellet. The lysates were sonicated for 15 s and then centrifuged for 10 min at 13,000× *g* and 4 °C. Protein concentration was measured using the Bradford method. A standard curve was generated on a black 96-well plate, and samples were applied according to the manufacturer’s protocol. Fluorescence was read after 30 min of incubation in the dark at λ = 390 nm (excitation) and λ = 460 nm (emission) using a Fluoroscan Ascent FL microplate reader. Enzyme units per 1 mL of sample were calculated from the linear regression equation and normalized to mg of protein.

### 4.10. Immunofluorescence and Immunocytochemistry Staining

The cells (1.5 × 10^4^ cells/well) were cultured for 24 h on ten-chambered glass slides (Greiner Bio-One, Frickenhausen, Germany) and then treated with the compounds for 48 h. Next, the cells were fixed with paraformaldehyde (4% *w*/*v* in PBS) and incubated with blocking buffer for 60 min (PBS/5% normal goat serum/0.3% Triton X-100), followed by incubation with primary antibodies against caspase-3 (cat.: D3RGY, at 1:400), phospho-histone H2AX(Ser139) (cat.: 9718, at 1:400) prepared in Antibody Dilution Buffer (PB/1% BSA/0.3% Triton X-100), and rabbit anti-RAD51 (Sigma-Aldrich, St. Louis, MO, USA, cat.: ABE257, at 1:500) monoclonal antibodies diluted with 1% BSA and 0.3% Triton X-100 in DPBS 1X. The secondary antibodies used were anti-rabbit IgG (H + L), F(ab’)2 fragment (Alexa Fluor 555 conjugate, at 1:1000) to detect caspase-3 and anti-rabbit IgG (H + L), F(ab’)2 fragment (Alexa Fluor 488 conjugate, at 1:2000) to detect phospho-histone H2AX. Nuclei were stained with 300 nm DAPI for 3 min at RT, rinsed once with DPBS 1X, and mounted with DPBS 1X for imaging. The excitation and emission parameters used were 405 nm and 460–480 nm, respectively. Immunofluorescence images were acquired using a Leica SP8 confocal microscope (Leica Microsystems, Germany), equipped with an environmental chamber (Okolab, Italy).

### 4.11. Caspase 3/7 Assay

The activities of caspases 3 and 7 were estimated with CellEvent™ Caspse-3/7 Green Detection Reagent (Thermo Fisher Scientific, Waltham, MA, USA) according to the manufacturer’s protocol. The cells were seeded on 96-well plates (15 × 10^3^/well), and after 24 h, they were incubated with the appropriate drugs for 24 or 48 h. The cells were fixed with a 4% paraformaldehyde solution for 10 min at room temperature (RT). The cells were labeled with CellEvent™ Caspase-3/7 Green Detection Reagent (5 μM) diluted in PBS with 5% FBS to avoid fluorescence background. After activation of caspase 3/7 in the apoptotic cells, the four-amino-acid (Asp-Glu-Val-Asp, DEVD) peptide was cleaved, enabling the dye to bind to DNA, which produced a bright fluorogenic response with absorption/emission maxima of 502/530 nm according to the manufacturer’s protocol. Fluorescence intensity was measured using a GloMax^®^ Explorer multimode microplate reader (Promega, Madison, WI, USA). Cysteine protease activity was expressed as a ratio of the fluorescence of drug-treated samples to that of the corresponding untreated controls (taken as 100%).

### 4.12. Olaparib-Resistant PDX Models of HGSOC

Immunodeficient NSG/J female mice were obtained from the Jackson Laboratory and maintained in a specific pathogen-free facility. To establish PDX models of HGSOC, pieces of fresh OC samples (10–20 mm^3^), obtained after surgical resection, were transplanted subcutaneously into both flanks of the NSG/J mice at The Maria Sklodowska-Curie National Research Institute of Oncology. The animals received daily (5 days a week) intraperitoneal (i.p.) injections of either olaparib (50 mg/kg) or the vehicle. Tumors that expanded their final size by at least 2-fold compared to their size at the start of treatment in the presence of olaparib for more than four weeks were considered resistant to olaparib. Cryopreserved fragments of olaparib-resistant tumors (~20 mm^3^) generated from PDX X179 were thawed and expanded through subcutaneous retransplantations into new recipient mice. Subsequently, after an increase in tumor volume to more than ~50 mm^3^, the animals were randomized into groups: vehicle control, olaparib (50 mg/kg), CHK1i (50 mg/kg), and olaparib + CHK1i (50 mg/kg + 50 mg/kg). The mice received a vehicle by i.p. injections five days a week, olaparib by i.p. injections five days a week, or CHK1i by i.p. injections twice a week. The animals were euthanized after 23 to 37 days of treatment (median 35 days) to evaluate treatment efficacy. Then, liver tissues were excised and dissected into pieces for analysis. The detailed procedure for this experiment has been described previously [16].

### 4.13. Preparation of Liver Sections and Immunohistochemistry

Tissue sections were prepared from harvested livers after ovarian cancer therapy. The tissue fragments were stored at −80 °C. The tissues were cut into 5 µm thick pieces and placed on positively charged glass slides (cryostat CM1950, Leica Biosystems). The preparations were fixed with 4% formaldehyde (15 min, RT) and then stained. The slides were incubated for 5 min with a Blocker of Endogenous Peroxidase (Bloxall Blocking Solution), (Vector Laboratories, Biokom, Poland). Subsequently, the slides were incubated for 1 h at RT with the following primary antibodies: anti-pCHK1 (Ser345) diluted 1:1000 or anti-Ki-67 diluted 1:200. Then, the slides were rinsed and incubated with SignalStain^®^ Boost Detection Reagent (HRP) (Cell Signaling) (Cell Signaling Technology Massachusetts, Danvers, MA, USA) for 30 min at RT. Staining was visualized by SignalStain^®^ DAB Chromogen (Cell Signaling) for 5 min. Nuclear contrast was achieved with hematoxylin counterstaining. Finally, the samples were mounted with DPX Mounting Medium (Merck). Afterward, cellular morphology changes were imaged at 20× magnification using an optical microscope (MICA WideFocal Live Cell, Leica Microsystems). Preparation and staining of the ovarian sections were described previously [16].

### 4.14. Hematoxylin and Eosin (H&E) Staining of Liver Tissue

The HE staining was performed with a Hematoxylin and Eosin Stain Kit (Vector Laboratories, Biokom, Poland) according to the manufacturer’s protocol. The samples were incubated in hematoxylin solution for 5 min, followed by rinsing in distilled water to remove the excess dye. The liver tissues were covered in Bluing Reagent and incubated for 10–15 s. Next, the slides were dipped in 100% ethanol (10 s) and incubated with eosin solution for 2–3 min. The probes were dehydrated in 3 changes of 100% ethanol (1–2 min each). Finally, the samples were mounted with DPX Mounting Medium (Merck) and subjected to a histological analysis. Afterward, cellular morphology changes were imaged at 20× magnification using an optical microscope (MICA WideFocal Live Cell, Leica Microsystems).

### 4.15. Statistical Analysis

The results are presented as mean ± SD from three independent studies. Statistica software, version 10.0 (StatSoft, Tulsa, OK, USA) was used for the statistical analysis. When comparing more than two groups, one-way analysis of variance (ANOVA) was performed and Tukey’s post hoc multiple comparisons test (MTT metabolic activity test; resazurin reduction test; Western blot results; and analysis of relative alanine aminotransferase, glutathione S-transferase, and caspase 3/7 activities) was used. The results on the oxidative stress analysis using MitoSOX Red and H2DCF-DA probes were statistically analyzed using two-factor ANOVA and Tukey’s post hoc test. Results with *p* < 0.05 were considered statistically significant.

## 5. Conclusions

The combination of targeted therapies presents promising avenues for improving outcomes in solid tumors. In this study, we explored novel therapeutic strategies for advanced HCCs in vitro. Additionally, we assessed the absence of liver metastases in vivo following initial ovarian cancer treatment in PDX models. The co-administration of CHK1 inhibitor and olaparib demonstrated a synergistic reduction in cell viability and an increase in DNA damage in HCC cells. In combination therapy, CHK1i impaired Rad51 foci formation induced by olaparib, while the induction of apoptosis varied across cell lines. Our results emphasize the importance of developing personalized treatment strategies to minimize the risks of overtreatment or suboptimal therapy. This study supports further evaluation of the therapeutic potential of combination treatment with CHK1i and PARPi for *BRCA*-mutated liver cancer and potential utility in solid tumor treatments such as ovarian cancer that metastasize to the liver.

## Data Availability

The data presented in this study are available from the corresponding author upon reasonable request.

## Supplementary Materials

The following supporting information can be downloaded at https://www.mdpi.com/article/10.3390/ijms26020834/s1.
