# Supplementary material for: MK-8776 and Olaparib Combination Acts Synergistically in Hepatocellular Carcinoma Cells, Demonstrating Lack of Adverse Effects on Liver Tissues in Ovarian Cancer PDX Model
Source: Int J Mol Sci. 2025 Jan 20;26(2):834. doi: 10.3390/ijms26020834 (PMC11766115; doi:10.3390/ijms26020834)
Supplement: Supplementary file 1 [file ijms-26-00834-s001.zip › Supplementary Information W. Bębenek.pdf]

# Supplementary Information

# MK-8776 and Olaparib Combination Act Synergistically in Hepatocellular Carcinoma Cells Demonstrating Lack of Adverse Effects on Liver Tissues in Ovarian Cancer PDX Model

**Wiktoria Bębenek, Arkadiusz Gajek, Agnieszka Marczak, Jan Malý, Jiří Smejkal, Małgorzata Statkiewicz, Natalia Rusetska, Magdalena Bryś and Aneta Rogalska** *

## Supplementary Figure S1 (Figure S1)

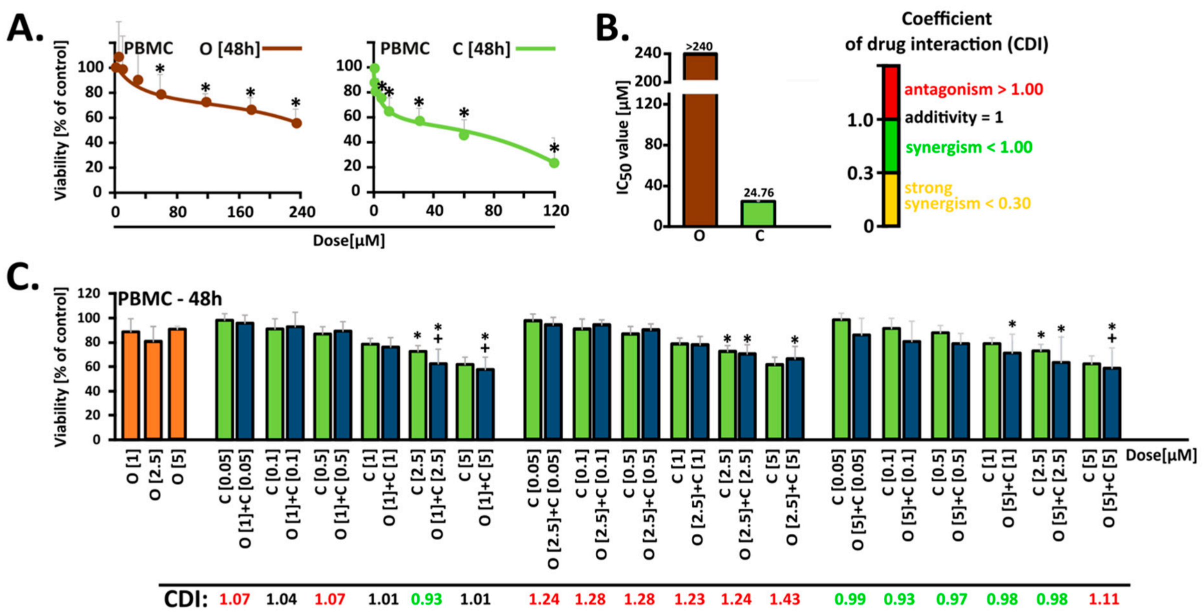


**Figure S1. (A)** Cell viability in response to five-day treatment with Olaparib (O, 1–240 μM) and CHK1i (C, 0.05–120 μM) was determined using the resazurin reduction assay in PMBCs cells after 48 hours incubation. **(B)** The $IC_{50}$ values. Data are expressed as mean ± SD (n ≥ 3). **(C)** Cytotoxic effects observed in PBMCs cells (48 hours) after treatment with O (1, 2.5 or 5μM) combined with C (0.05, 0.1, 0.5, 1, 2.5 and 5 μM). Data are expressed as mean ± SD (n = 3–6). Coefficient of drug interaction (CDI) values indicating whether interaction effects are significantly synergistic (CDI < 1), additive (CDI = 1.0), or antagonistic (CDI > 1.0). Statistical significance was assessed using ANOVA followed by Tukey's test. * Statistically significant changes between cells treated with the compound and control cells ($p < 0.05$). + Statistically significant changes between cells treated with PARPi and combination treatments (O + C) ($p < 0.05$). # Statistically significant changes between cells treated with CHK1i and combination treatments (O + C) ($p < 0.05$).

## Supplementary Figure S2 (Figure S2)

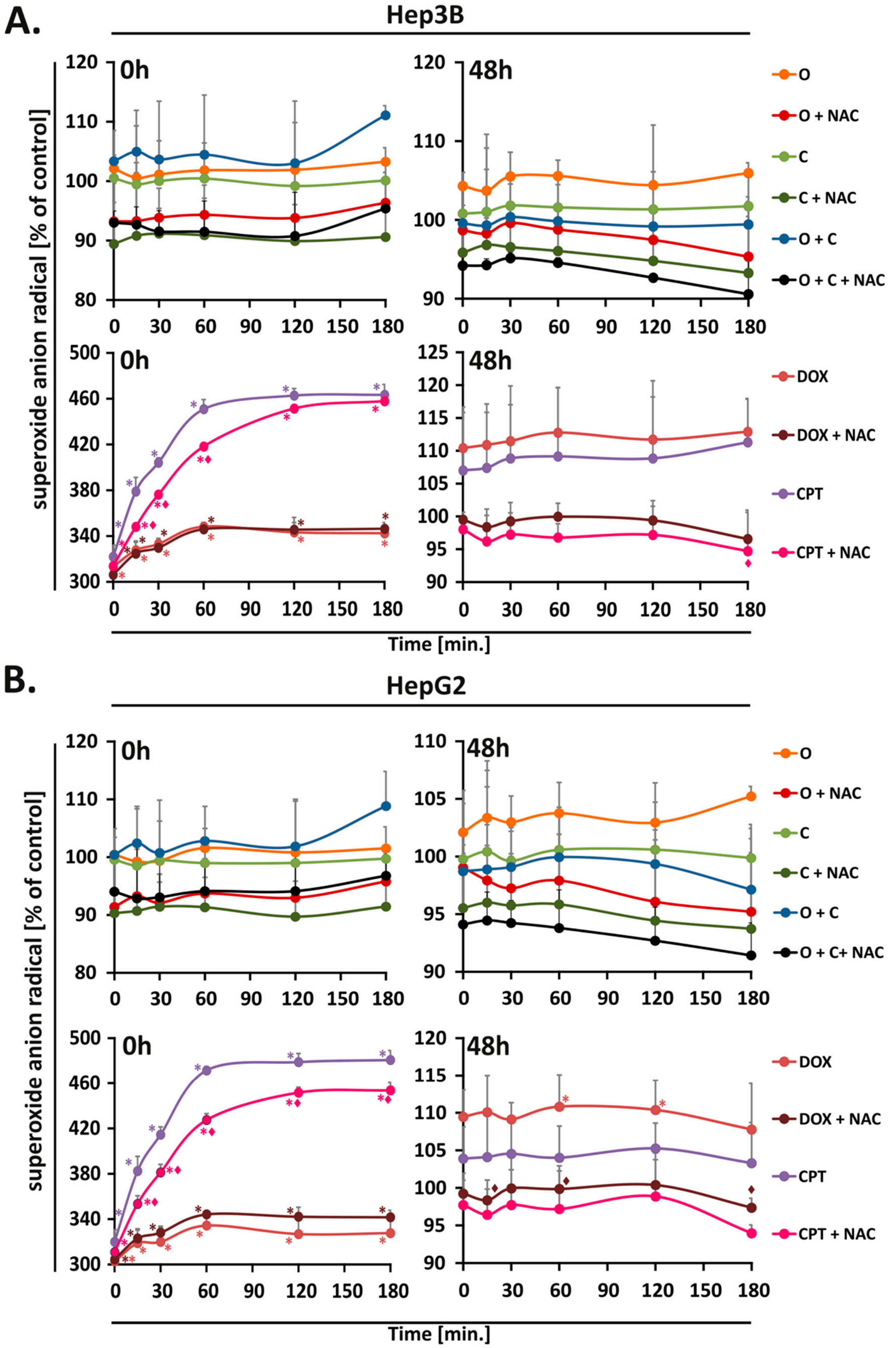

**Figure S2.** Mitochondrial ROS activity. The kinetics of ROS generation in Hep3B (A) and HepG2 (B) cells after treatment with O (2.5 μM), C (5 μM) and the combination of O + C (2.5 μM; 5 μM) were measured immediately after adding the drugs up to 180 minutes, and after 48 hours up to 180 minutes in the presence or absence of an antioxidant (NAC). The control cells (not treated) were assumed as 100%. Positive controls cells were treated with doxorubicin, (DOX, 10 μM) or camptothecin, (CPT, 5μM). Data are expressed as mean ± SD ($n$ = 3–6). Statistical significance was assessed using ANOVA followed by Tukey's test. * Statistically significant changes between samples incubated with the compound compared with control cells ($p < 0.05$). ◊ Statistically significant changes between samples incubated with DOX aor CPT and samples preincubated with N-acetylcysteine ($p < 0.05$).

## Supplementary Table (S1)

**Key materials and reagents used in the study**

| Reagent | Catalogue Number | Manufacturer |
|---|---|---|
| 0.5 M EDTA | 78442 | Thermo Fisher Scientific |
| Blocker of Endogenous Peroxidase | VC-SP-6000 | Vector Laboratories |
| BSA (bovine serum albumin) | A3059 | Sigma-Aldrich (Merck) |
| Camptotecin | C9911 | Millipore (Merck) |
| DCFH2-DA | D6883 | Sigma-Aldrich (Merck) |
| DMEM, high glucose, GlutaMAX™ Supplement, HEPES | 32430100 | Gibco (Thermo Fisher Scientific) |
| DMSO (dimethyl sulfoxide) | 363550117 | Avantor Performance Materials Poland |
| Doxorubicin | D1515 | Millipore (Merck) |
| DPX Mounting Medium | 6522 | Sigma-Aldrich (Merck) |
| FBS, heat-inactivated, qualified | A5256801 | Gibco (Thermo Fisher Scientific) |
| H2O2 | 21;676-3 | Sigma-Aldrich (Merck) |
| HaltTM Protease & Phosphatase Single-Use Inhibitor Cocktail (100x) | 78442 | Thermo Fisher Scientific |
| Lymphosep (Lymphosep Separation Media) | L0560500 | Biowest LLC |
| Methanol >99% | BA1990110 | Avantor Performance Materials Poland |
| MitoSOXTM Red mitochondrial superoxide indicator | M36008 | Invitrogen (Thermo Fisher Scientific) |
| MOPS SDS Running Buffer | MPM0PS | Millipore (Merck) |
| mPAGE® 4X LDS Sample Buffer | MPSB | Millipore (Merck) |
| mPAGE® Bis-Tris Precast Gels 10% | MP10W10 | Millipore (Merck) |
| MTT | 20395.03 | SERVA Electrophoresis |
| NAC (N-acetylcysteine) | A7250-10G | Sigma-Aldrich (Merck) |
| PBS (phosphate buffered saline) | X0515 | Biowest LLC |
| PerceTM Detergent Compatible Bradford Assay Reagent | 1863028 | Thermo Fisher Scientific |
| PHA-M (phutohemagglutinin) | L3010005 | Biowest LLC |
| PMSF (phenylmethylsulfonyl fluoride) | 36078 | Thermo Fisher Scientific |
| PVDF Membrane | IPVH85R | Millipore (Merck) |
| Resazurin | R7017 | Sigma-Aldrich (Merck) |

| | | |
|---|---|---|
| RIPA Lysis and Extraction Buffer | 89900 | Thermo Fisher Scientific |
| RPMI 1640 Medium, GlutaMAX™ Supplement, HEPES | 72400054 | Gibco (Thermo Fisher Scientific) |
| RPMI 1640 Medium, with L-Glutamine, without Phenol Red | E15848 | PAA Laboratories GmbH |
| SignalStain® Boost Detection Reagent | 8114S | Cell Signaling Technology |
| SignalStain® DAB Chromogen | 72986S | Cell Signaling Technology |
| SpectraTM Multicolor High Range Protein Ladder | 26625 | Thermo Fisher Scientific |
| SuperSignalTM West Pico PLUS Chemiluminescent Substrate | 34580 | Thermo Fisher Scientific |
| Transfer Buffer | MPTRB | Millipore (Merck) |
| Trypsin-EDTA | 25200072 | Gibco (Thermo Fisher Scientific) |
| β-mercaptoethanol | M6250 | Sigma-Aldrich (Merck) |

## Supplementary Table (S2)

**Tris Buffered Saline with Tween 20**

| **Tris Buffered Saline with Tween 20 (Western Blotting)** | | |
|---|---|---|
| HCL | H1758 | Sigma-Aldrich (Merck) |
| NaCl | 71376 | Sigma-Aldrich (Merck) |
| Tris Base | 93362 | Sigma-Aldrich (Merck) |
| Tween 20 | P1379 | Sigma-Aldrich (Merck) |

## Supplementary Table (S3)

**Lysis Buffer (PBMCs)**

| **Lysis Buffer** |
|---|
| 150 mM $NH_4Cl$ [53.49 g/mol] |
| 10 mM $NaHCO_3$ [84.01 g/mol] |
| 1 mM EDTA [372.40 g/mol] |

## Supplementary Table (S4)

**Assay Kits used in the study**

| **Assay Kit** | | |
|---|---|---|
| Alanine Transaminase Activity Assay kit (Colorimetric/Fluorometric) | ab105134 | Abcam |
| Hematoxylin and Eosin Stain Kit | VC-H-3502 | Vector Laboratories |
| Glutathione S-Transferase Fluorescent Activity Kit | EIAGSTF | Invitrogen (Thermo Fisher Scientific) |
| CellEvent™ Caspse-3/7 Green Detection Reagent | C10723 | Invitrogen (Thermo Fisher Scientific) |

**<u>Supplementary Table (S5)</u>**

**Primary antibodies used in the study**

| Target | Host and Clonality | Manufacturer | Catalogue Number | Dilution | Dilution buffer | Blocking agent |
|---|---|---|---|---|---|---|
| caspase-3 | Rabbit monoclonal | Cell Signaling Technology | D3RGY | 1:400 | 5% BSA in TBST | Blocking Buffer |
| γH2AX (Ser139) | Rabbit monoclonal | Cell Signaling Technology | 9718S | 1:1000 (WB),1:400 (IF) | 5% non-fat milk in TBST | 5% non-fat milk in TBST, Blocking Buffer |
| β-actin | Mouse monoclonal | Sigma-Aldrich (Merck) | A1978 | 1:10000 | 5% non-fat milk in TBST | 5% non-fat milk in TBST |
| RAD51 | Rabbit monoclonal | Sigma-Aldrich (Merck) | ABE257 | 1:500 | 5% BSA in TBST | Blocking Buffer |
| phospho-CHK1 (Ser345) | Rabbit monoclonal | Invitrogen (Thermo Fisher Scientific) | PA5-34625 | 1:1000 | 5% non-fat milk in TBST | Blocking Buffer |
| PARP1 | Rabbit monoclonal | Cell Signaling Technology | 9532S | 1:1000 | 5% non-fat milk in TBST | 5% non-fat milk in TBST |
| Ki-67 | Rabbit monoclonal | Invitrogen (Thermo Fisher Scientific) | MA5-14520 | 1:200 | 5% non-fat milk in TBST | Blocking Buffer |

## Supplementary Table (S6)

**Secondary antibodies used in the study**

| Species Reactivity | Conjugate | Host | Manufacturer | Catalogue Number | Dilution | Dilution buffer |
|---|---|---|---|---|---|---|
| Mouse | Alexa Fluor™ Plus 488 | Goat | Invitrogen (Thermo Fisher Scientific) | A32723 | 1000 | DPBS 1X with 1% BSA and 0.3% Triton X-100 |
| Mouse | HRP | Goat | Invitrogen (Thermo Fisher Scientific) | A28177 | 1:10000 | 5% non-fat milk in TBST |
| Rabbit | HRP | Goat | Cell Signaling Technology | 7074 | 1:3000 | 5% non-fat milk in TBST |
| Rabbit | Alexa Fluor™ Plus 555 | Goat | Cell Signaling Technology | 4413 | 2000 | ŌDPBS 1X with 1% BSA and 0.3% Triton X-100 |

## Supplementary Figure S3 (Figure S3)

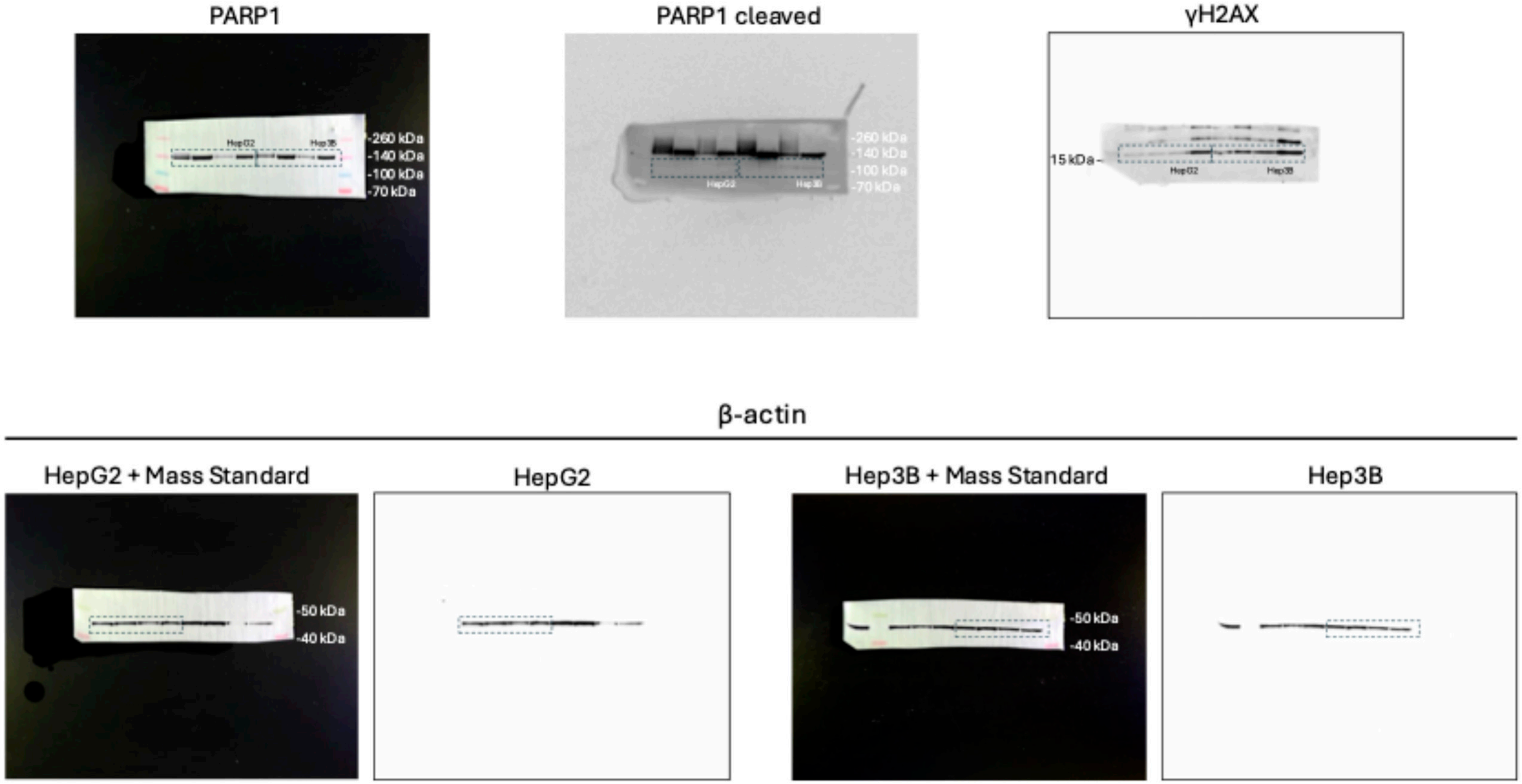


**Figure S3. Original photos of membranes.**
