# Supplementary figures and images for: MK-8776 and Olaparib Combination Acts Synergistically in Hepatocellular Carcinoma Cells, Demonstrating Lack of Adverse Effects on Liver Tissues in Ovarian Cancer PDX Model
Source: Int J Mol Sci. 2025 Jan 20;26(2):834. doi: 10.3390/ijms26020834 (PMC11766115; doi:10.3390/ijms26020834)

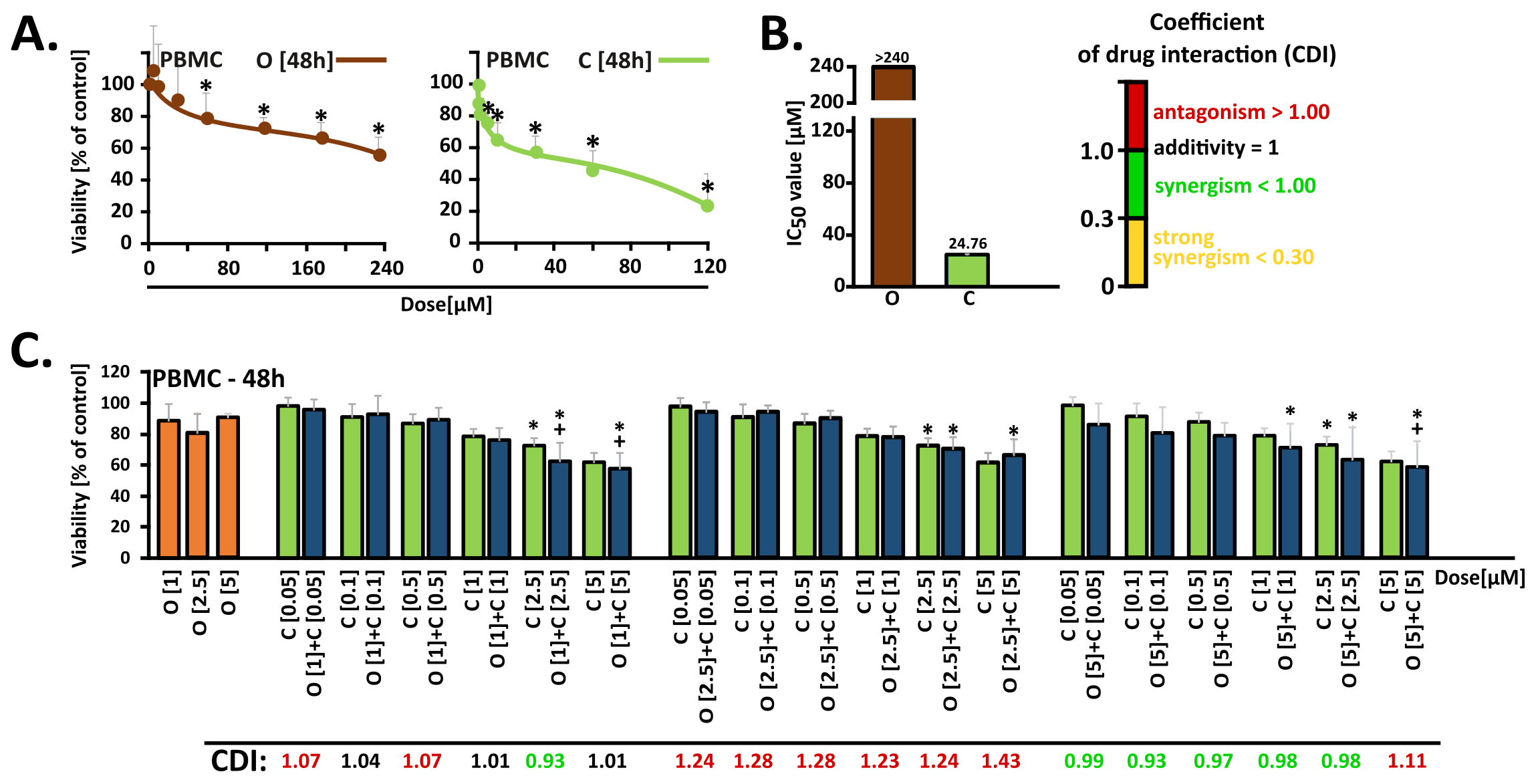

Supplement: Supplementary file 1 [file ijms-26-00834-s001.zip › Fig. 1S.png]

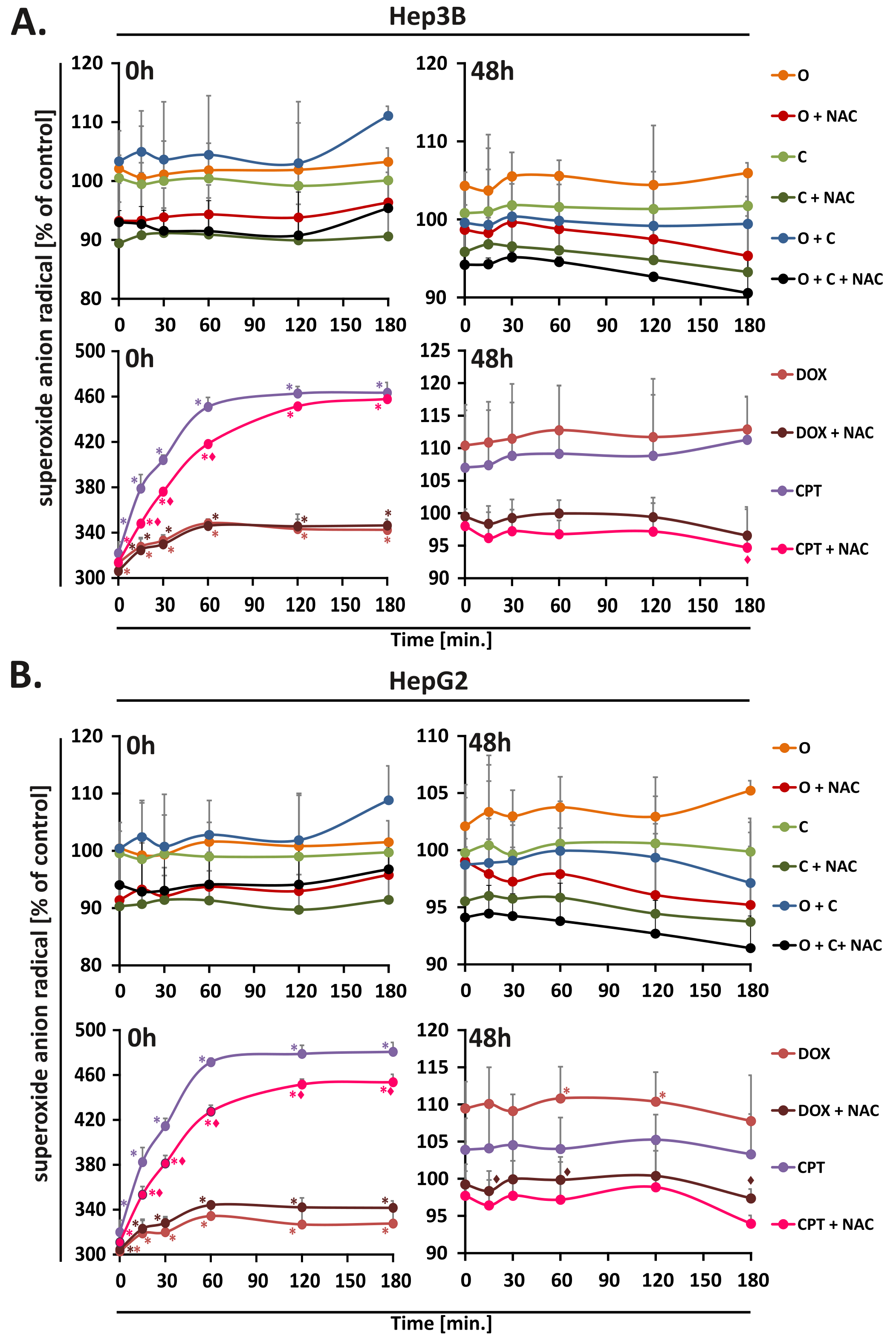

Supplement: Supplementary file 1 [file ijms-26-00834-s001.zip › Fig. 2S.png]

PARP1

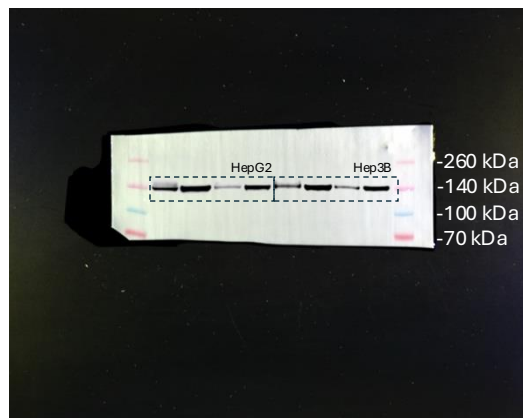

PARP1 cleaved

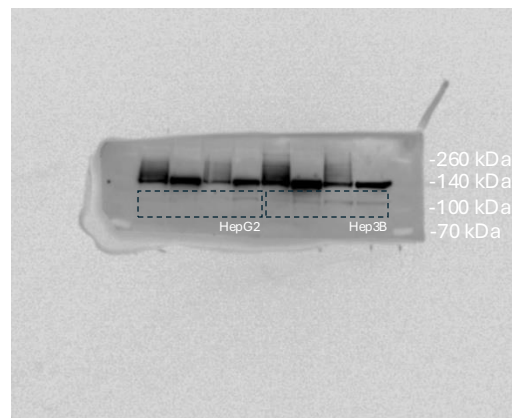

$\gamma$ H2AX

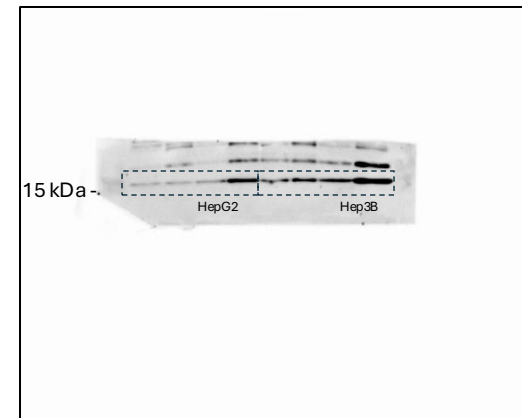

$\beta$ -actin

HepG2 + Mass Standard

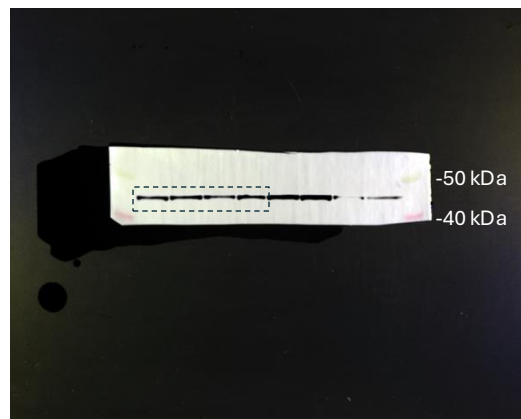

HepG2

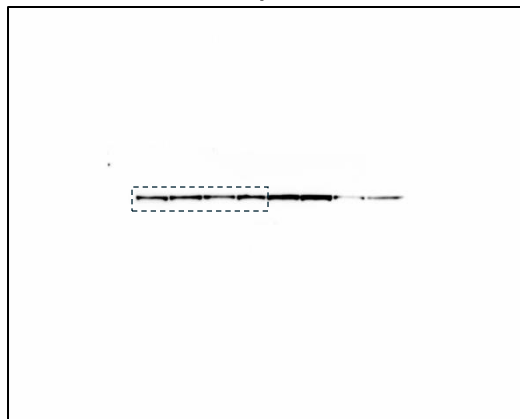

Hep3B + Mass Standard

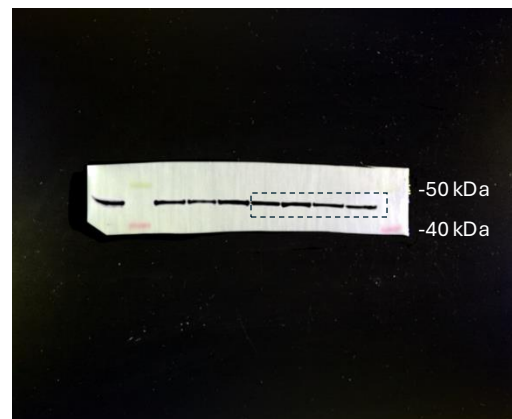

Hep3B

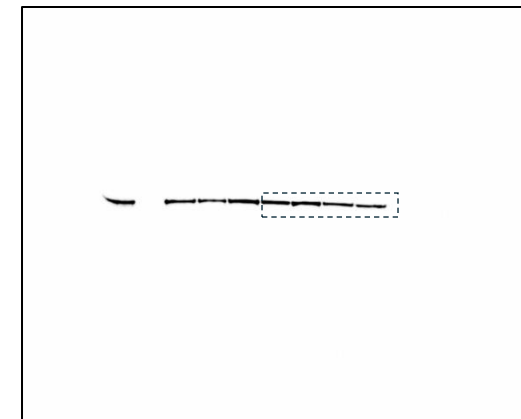

Supplement: Supplementary file 1 [file ijms-26-00834-s001.zip › Fig. 3S.pdf]
